# Starch Degradation and Soluble Sugar Accumulation Are Associated with Cold Tolerance in Winter Rapeseed (*Brassica napus* L.)

**DOI:** 10.3390/plants15162534

**Published:** 2026-08-21

**Authors:** Lin Long, Yangyang Shi, Yuanyuan Pu, Junyan Wu, Li Ma, Lijun Liu, Gang Yang, Tingting Fan, Wangtian Wang, Wancang Sun

**Affiliations:** 1 College of Agronomy, Gansu Agricultural University, Lanzhou 730070, China; 1073324120563@st.gsau.edu.cn (L.L.); syy409287@163.com (Y.S.); wujuny@gsau.edu.cn (J.W.); yangang1018@163.com (G.Y.); fantt@gsau.edu.cn (T.F.); 18293121851@163.com (W.S.); 2 Seed Industry Research Institute of Gansu Provincial University, Lanzhou 730070, China; 3 State Key Laboratory of Aridland Crop Science, Lanzhou 730070, China; mal@gsau.edu.cn (L.M.); liulj198910@163.com (L.L.); 4 College of Life Science and Technology, Gansu Agricultural University, Lanzhou 730070, China; wangw@gsau.edu.cn

**Keywords:** cold stress, winter rapeseed, leaf ultrastructure, starch and sugar metabolism, transcriptomic

## Abstract

Winter rapeseed suffers severe damage under cold stress, which seriously restricts its growth and yield. However, differences among cultivars in leaf starch and soluble sugar, as well as their underlying molecular regulatory mechanisms under low-temperature stress, remain poorly understood. We hypothesized that winter rapeseed cultivars with differences in cold tolerance would vary in starch accumulation, soluble sugar contents, and expression of the starch and sugar metabolism-related genes under cold stress. This study aimed to investigate differences in starch accumulation, soluble sugar content, and the gene expression patterns involved in the starch and sugar metabolism pathway in the leaves of winter rapeseed (*Brassica napus* L.) cultivars with contrasting cold tolerance under cold stress. In this study, two winter rapeseed cultivars, cold-tolerant L88 and cold-sensitive T2288, received 24 h cold treatments at 22 °C (control), 4 °C, 0 °C, and −4 °C. Plant morphology and leaf ultrastructure were observed, iodine–potassium iodide (I_2_-KI) staining was performed, and the starch, soluble sugar contents (sucrose, fructose, glucose, and maltose) were analyzed for each temperature regime. Transcriptome sequencing was performed exclusively at −4 °C (0 h versus 24 h). Under −4 °C stress, the two cultivars showed the greatest differences in plant phenotype and leaf ultrastructure, with the cold-sensitive cultivar Tianyou 2288 suffering more severe freezing injury. In Longyou 88, chloroplasts in mesophyll cells were swollen and contained fewer starch granules, whereas in Tianyou 2288 some chloroplasts disintegrated and large numbers of starch granules remained undegraded. As the treatment temperature decreased, leaf starch content declined in both cultivars. At −4 °C, starch content in Longyou 88 was 3.84 mg/g lower than in Tianyou 2288. The contents of soluble sugars, sucrose, glucose, and maltose in leaves increased continuously in both cultivars as stress temperature decreased and reached their maximum values at −4 °C. However, fructose content showed cultivar-specific changes. In Longyou 88, fructose content reached its maximum value of 12.59 mg/g at −4 °C, whereas in Tianyou 2288 it peaked at 7.54 mg/g at 4 °C. At −4 °C, fructose content was 6.69 mg/g higher in Longyou 88 than in Tianyou 2288. Transcriptome analysis revealed that after 24 h of −4 °C stress, Longyou 88 had more upregulated differentially expressed genes (DEGs) than Tianyou 2288, whereas the number of downregulated DEGs in Tianyou 2288 was approximately twice that in Longyou 88. The genes *BAM1*, *BAM3*, *SUS1*, *SCRK1*, *TPS9*, and *E1314*, which were involved in the starch and sucrose metabolism pathway, were upregulated in both cultivars, whereas *BGL44* was downregulated. These findings indicate that cold-tolerance winter rapeseed is associated with enhanced starch degradation, maintenance of chloroplast structural integrity in mesophyll cells, and increased soluble sugar accumulation, providing potential physiological indicators for cold-tolerance evaluation.

## 1. Introduction

Low temperature is one of the major environmental factors limiting plant growth, development, and geographical distribution throughout the plant life cycle [1]. During cold acclimation, plants undergo structural remodeling of organelles, particularly chloroplasts, and enhance membrane system stability, thereby improving their adaptation and resistance to low-temperature stress [2]. Chloroplasts are among the organelles most sensitive to low temperature, which can directly impair the integrity of chloroplast membrane systems, resulting in loose thylakoids organization, membrane disruption, and even chloroplast disintegration [3,4]. In mesophyll cells of camphor tree (*Cinnamomum camphora*) leaves, chloroplasts exhibited slight swelling at 5 °C and pronounced swelling and deformation at 0 °C, accompanied by increased numbers of osmiophilic granules and starch granules. At −10 °C, the chloroplast membrane system was completely disrupted, the stroma lamellae of thylakoids fragmented into filament-like structures, and some chloroplasts became hollow or completely disintegrated [5]. After treatment at 4 °C for 24 h, mesophyll cell structures in the cold-tolerant cassava cultivar ‘F200’ remained relatively intact, and chloroplasts maintained normal photosynthetic activity and continued starch synthesis. In contrast, the cold-sensitive cultivar ‘South China 8’ exhibited cell wall damage and abnormal starch accumulation within chloroplasts [6]. Under −2 °C stress, the double-membrane structure of loquat chloroplasts was severely damaged, and thylakoid stacking was observably reduced. When the temperature decreased to −7 °C, the leaves exhibited severe freezing injury, chloroplasts were completely disintegrated, and the grana lamellar became disorganized [7]. Chloroplasts are the major site for starch synthesis and soluble-sugar conversion in leaf cells, and their structure is highly susceptible to low-temperature damage. Cold-induced chloroplast injury impairs photosynthetic carbon assimilation and disturbs carbohydrate metabolism [8]. Further, starch degradation and subsequent soluble sugar accumulation within chloroplasts may mitigate freezing damage by supporting osmotic adjustment and energy supply [9]. Such interactions may suggest a close correlative relationship between chloroplast structural stability and starch–sugar dynamics under cold stress, and this coordination pattern frequently differs among cultivars with different cold-tolerance performance.

Starch serves as an important energy reserve in plants and plays a crucial role in plant adaptation to abiotic stresses, including low temperature [10]. Previous studies have shown that in evergreen leaves, starch content decreases as temperatures decline in autumn, whereas soluble sugar content increases significantly. This increase in soluble sugars is closely associated with enhanced cold tolerance in plants [11,12,13]. Low temperature can promote starch hydrolysis and increase soluble sugar content. When temperatures subsequently rise, soluble sugars in the cytoplasm can be reconverted into starch and stored stably within cells. These changes suggest that the dynamic conversion between starch and soluble sugars plays an important role in plant responses to low-temperature stress [14,15]. Soluble sugars are important osmotic regulators in plant cells and mainly include sucrose, fructose, glucose, maltose, and other low-molecular-weight carbohydrates [16]. Under low-temperature conditions, *β*-amylase (BAM) promotes starch degradation in plant leaves, thereby contributing to the accumulation of sucrose, maltose, and other soluble sugars [17]. In *Medicago falcata* [18], dormant buds withstand low-temperature stress during overwintering by increasing cellular soluble sugar concentrations, thereby lowering the freezing point and enhancing tolerance to low-temperature stress. During cold acclimation in tea plants, leaf starch content gradually decreases, whereas the contents of soluble sugars, sucrose, glucose, and maltose increase, thereby contributing to enhanced cold tolerance [19]. Many studies have reported shifts in leaf starch and sugar metabolism under cold stress. However, little is known about the transcriptional mechanisms that drive varied metabolic patterns across cultivars with contrasting cold stress. Transcriptome profiling provides a feasible approach to identify key genes regulating carbohydrate metabolism during freezing stress [8].

Winter rapeseed is an important overwintering oilseed and cover crop in northern China. However, cold stress remains a major factor limiting its large-scale cultivation in the cold and arid regions of northern China [20]. In this study, we used cold-tolerant Longyou 88 and cold-sensitive Tianyou 2288 and exposed plants to 22 °C, 4 °C, 0 °C, and −4 °C for 24 h. Phenotype, physiology, and chloroplast ultrastructure were examined across all treatments, whereas transcriptomic profiling was performed only at −4 °C (0 h versus 24 h). Relative to Tianyou 2288, Longyou 88 maintained better chloroplast integrity under cold stress, accumulated more soluble sugars while starch declined, and showed stronger upregulation of starch and sucrose metabolism genes. *BAM1*, *BAM3* and *SUS1* emerged as candidate genes underlying the cultivar difference in cold tolerance. The aim was to identify potential indicators of cold tolerance and provide a theoretical basis for the screening and breeding of cold-tolerant winter rapeseed cultivars suitable for northern China.

## 2. Results

### 2.1. Effects of Low-Temperature Stress on Seedling Phenotypes of Winter Rapeseed Cultivars with Contrasting Cold Tolerance

**Figure 1:**
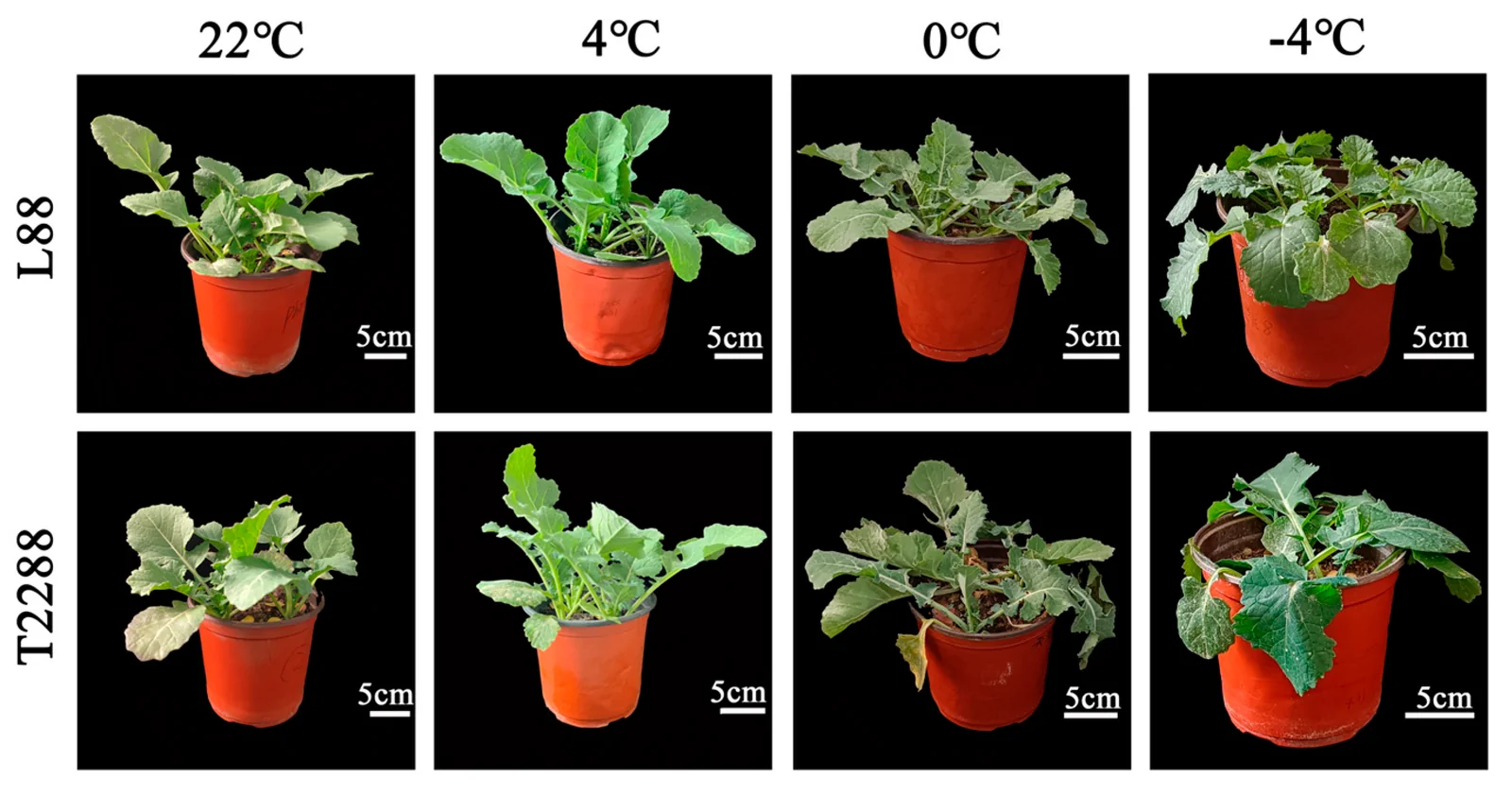
Phenotypic changes in winter rapeseed (*B. napus*) cultivars with contrasting cold tolerance under cold stress. Seedlings were exposed to 22 °C (control), 4 °C, 0 °C, and −4 °C for 24 h, and photographs were taken immediately after cold stress treatment. Scale bar = 5 cm.

Morphological changes under low-temperature stress provide a direct indication of the extent of stress-induced injury in plants. As shown in Figure 1, no obvious phenotypic differences from the control were observed in either cultivar following exposure to 4 °C for 24 h. After treatment at 0 °C for 24 h, several older leaves of the cold-tolerant cultivar L88 showed slight wilting, whereas those of the cold-sensitive cultivar T2288 exhibited more pronounced dehydration and wilting (Figure 1). Following freezing stress at −4 °C for 24 h, leaves of both cultivars drooped, and water-soaked, translucent symptoms of freezing injury were observed in both petioles and leaf blades (Figure 1). These symptoms were more severe in T2288 than in L88. Collectively, no obvious phenotypic differences were observed between the two cultivars under 4 °C stress, whereas T2288 exhibited more severe injury than L88 under 0 °C and −4 °C stress.

### 2.2. Changes in Leaf Ultrastructure of Winter Rapeseed Cultivars with Contrasting Cold Tolerance Under Low-Temperature Stress

**Figure 2:**
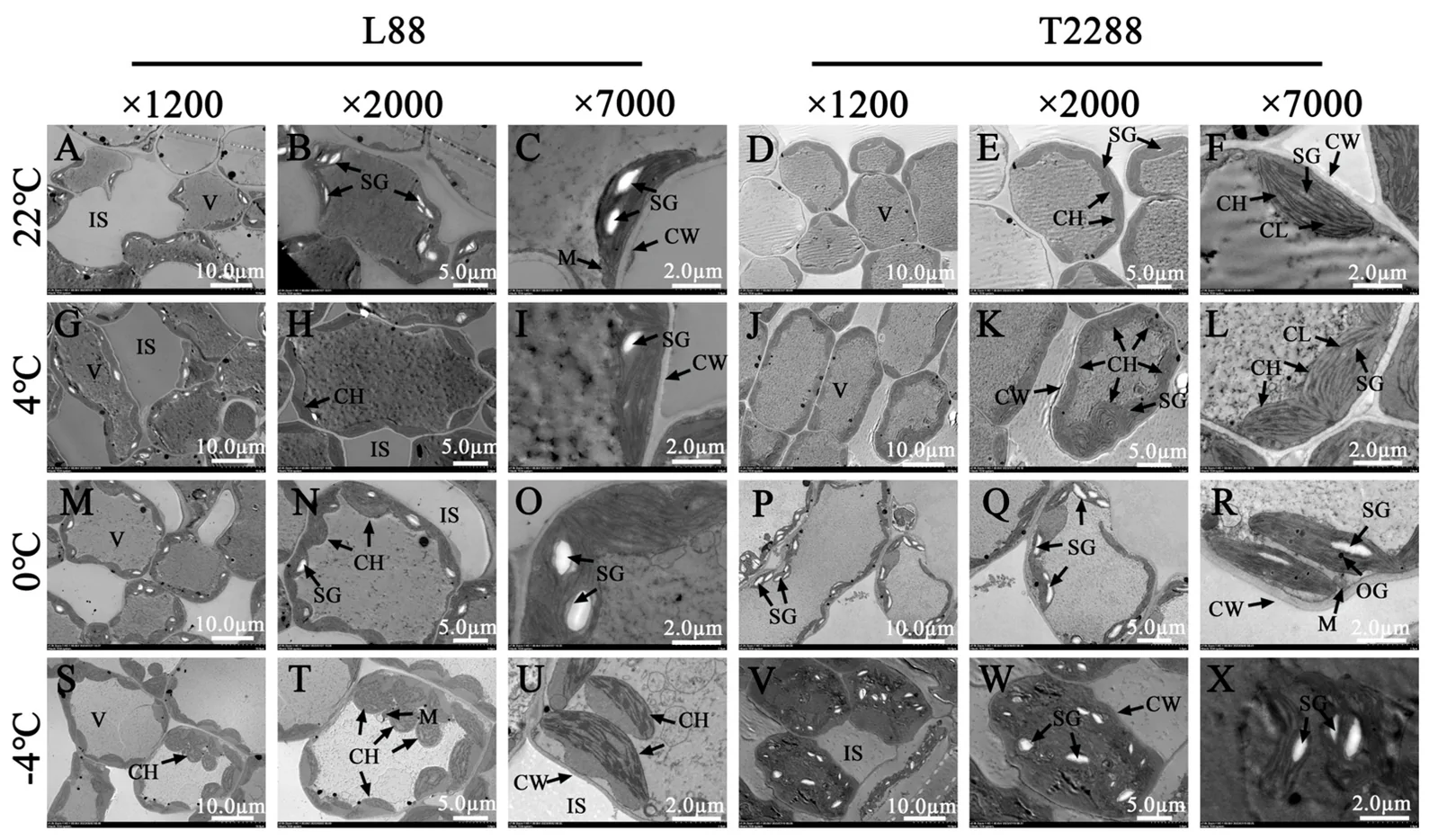
Ultrastructural changes in leaf mesophyll cells between winter rapeseed *(B. napus*) cold-tolerant L88 and cold-sensitive winter rapeseed (*B. napus*) T2288 under cold stress. (**A**–**C**,**G**–**I**,**M**–**O**,**S**–**U**): cold-tolerant cultivar L88 under 22 °C, 4 °C, 0 °C, and −4 °C, respectively; (**D**–**F**,**J**–**L**,**P**–**R**,**V**–**X**): cold-sensitive cultivar T2288 under 22 °C, 4 °C, 0 °C, and −4 °C, respectively. For each temperature, three magnifications are displayed: ×1200 (**A**,**D**,**G**,**J**,**M**,**P**,**S**,**V**), ×2000 (**B**,**E**,**H**,**K**,**N**,**Q**,**T**,**W**), ×7000 (**C**,**F**,**I**,**L**,**O**,**R**,**U**,**X**). Scale bars are indicated in each panel. Abbreviations: CH: chloroplasts; SG: starch granules; CW: cell wall; V: vacuoles; M: mitochondria; IS: intercellular space; CL: chloroplast inner membrane lamellae; OG: osmiophilic granules.

Under control conditions, mesophyll cells of both cultivars displayed typical cellular ultrastructure and intact cell walls (Figure 2A–F). Chloroplasts were spindle-shaped or oval, closely appressed to the inner surface of the cell wall, and the chloroplast membranes were visible. The grana lamellae showed high electron density and orderly stacking, and starch granules were present within chloroplasts (Figure 2A–F). After exposure to 4 °C for 24 h, the cold-tolerant cultivar L88 showed little change in chloroplast morphology, number, or starch granule distribution compared with the control (Figure 2G–I). In the cold-sensitive cultivar T2288, however, a few chloroplasts showed slight swelling and became oval, with starch granules rarely detected, compared with the control (Figure 2J–L). After exposure to 0 °C for 24 h, both cultivars exhibited ultrastructural alterations to varying degrees (Figure 2M–R). In L88, chloroplasts were slightly swollen and contained numerous starch granules, whereas in T2288 the grana lamellae appeared blurred and disorganized although abundant starch granules were also present. Following exposure to −4 °C for 24 h, the ultrastructural differences between the two cultivars became more pronounced (Figure 2S–X). In L88, the cell wall remained intact and the plasma membrane was clearly discernible, although chloroplasts were more severely damaged than at 0 °C (Figure 2M–O,S–U). Notably, the number of starch granules decreased significantly. When compared under the same −4 °C cold-stress condition, T2288 exhibited severe organelle damage, characterized by chloroplast breakdown and degradation (Figure 2V–X). The organelles were aggregated toward the center of the cell, accompanied by substantial starch granule accumulation. These observations indicate that, under cold stress, chloroplast ultrastructure was less severely disrupted in the cold-tolerant cultivar L88 than in the cold-sensitive cultivar T2288.

### 2.3. Changes in Leaf Starch Accumulation in Winter Rapeseed Cultivars with Contrasting Cold Tolerance Under Cold Stress

**Figure 3:**
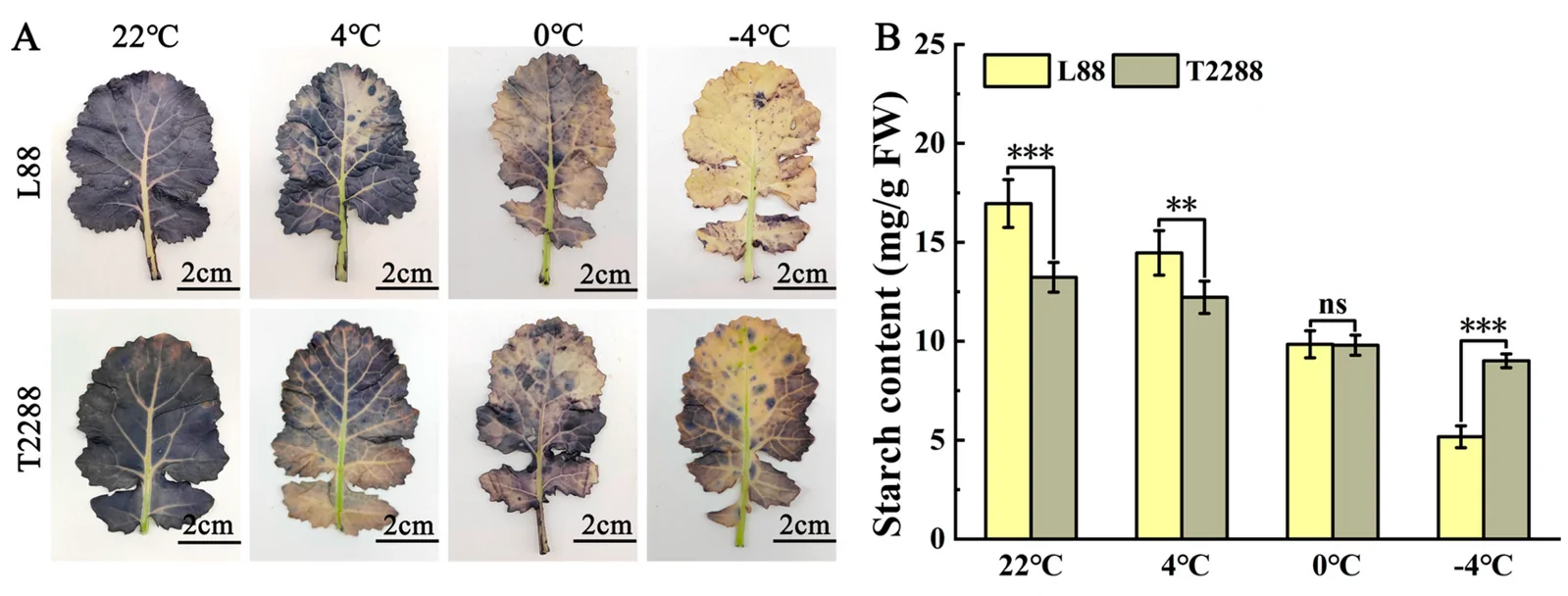
Starch accumulation in leaves of cold-tolerant winter rapeseed (*B. napus*) L88 and cold-sensitive winter rapeseed (*B. napus*) T2288 under cold stress. (**A**) I_2_–KI staining of leaves. Scale bar = 2 cm. (**B**) Quantitative starch content. Asterisks indicate significant differences, ns represents not significant (*p* ≥ 0.05), ** represents *p* ≤ 0.01, and *** represents *p* ≤ 0.001.

As the treatment temperature decreased, the I_2_–KI-stained area in leaves gradually diminished, accompanied by weaker staining intensity (Figure 3A). Starch contents showed the same trend (Figure 3B). Under control conditions, L88 exhibited a larger stained area and deeper coloration than T2288, with starch contents of 16.96 mg/g and 13.23 mg/g, respectively. Under 4 °C stress, the staining area and staining intensity were reduced in both cultivars compared control conditions, but L88 showed stronger staining than T2288 and a significantly higher starch content (14.46 mg/g vs. 12.22 mg/g). Under 0 °C treatment, no significant difference in starch content was detected between L88 and T2288 for this identical cold-stress treatment id condition (9.84 mg/g and 9.80 mg/g, respectively). After 24 h at −4 °C, both cultivars exhibited further reductions in stained area and staining intensity. Nevertheless, when comparing the two cultivar under this treatment, the starch content of L88 decreased to 5.17 mg/g, which was significantly lower than that of T2288 (9.01 mg/g). These results suggest that L88 exhibited greater starch degradation and lower residual starch accumulation than T2288 under cold stress.

### 2.4. Changes in Leaf Soluble Sugar Content in Winter Rapeseed Cultivars with Contrasting Cold Tolerance Under Cold Stress

**Figure 4:**
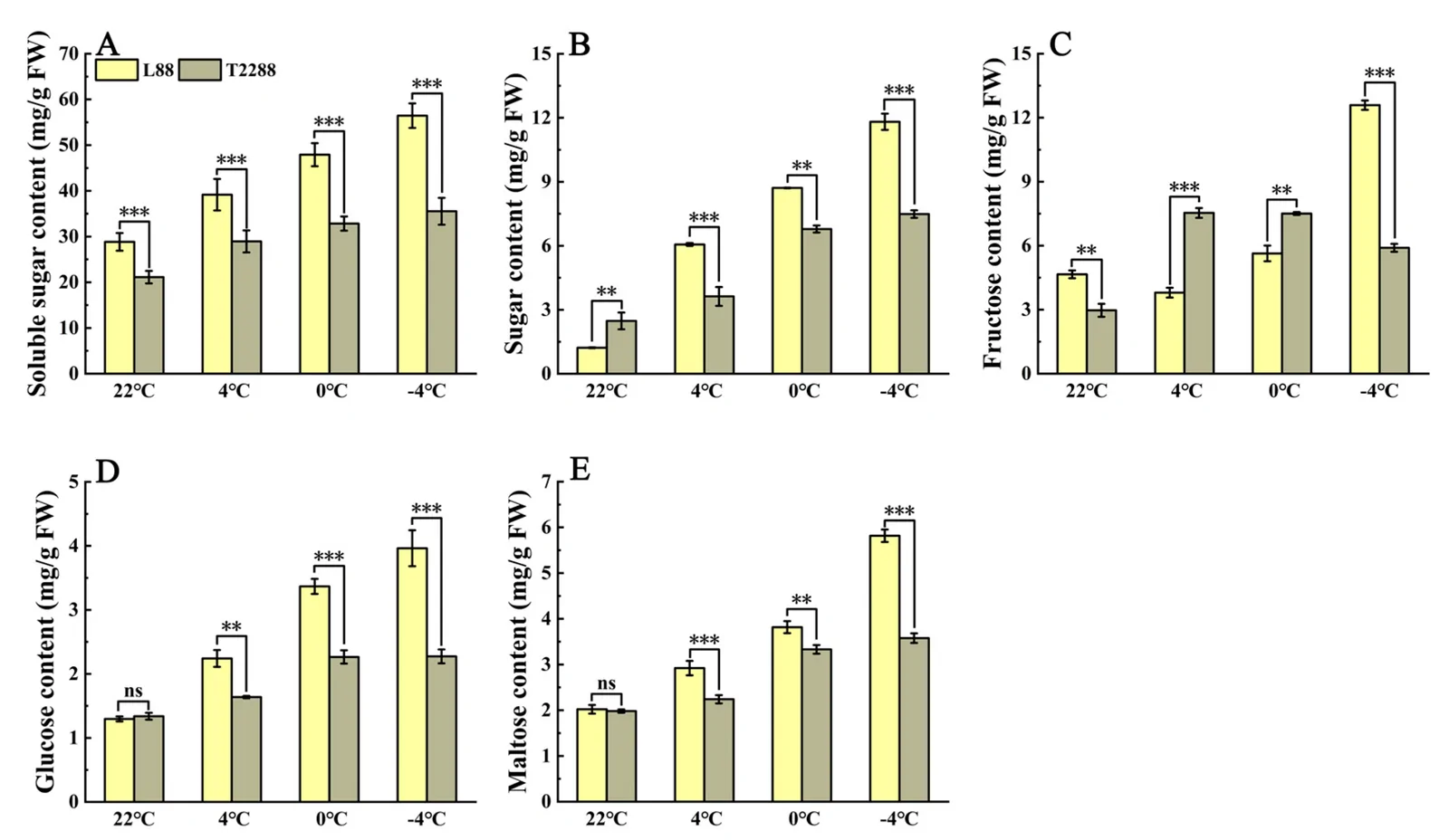
Changes in soluble sugar and individual soluble sugars content in leaves of cold-tolerant winter rapeseed (*B. napus*) L88 and cold-sensitive winter rapeseed (*B. napus*) T2288 under cold stress. (**A**) Soluble sugar content; (**B**) sucrose content; (**C**) fructose content; (**D**) glucose content; (**E**) maltose content. Yellow bars: L88; grey bars: T2288. Asterisks indicate significant differences, ns represents not significant (*p* ≥ 0.05), ** represents *p* ≤ 0.01, and *** represents *p* ≤ 0.001.

Soluble sugar content increased in the leaves of both cultivars as the treatment temperature decreased (Figure 4A). Across all treatments, the cold-tolerant cultivar L88 consistently maintained higher soluble sugar content than the cold-sensitive cultivar T2288. From 22 °C to −4 °C, soluble sugar content in L88 increased from 28.84 to 56.45 mg/g, corresponding to a 95.73% increase, whereas that in T2288 increased by 68.22%. Thus, soluble sugar accumulation increased in both cultivars under cold stress, with a more pronounced increase in L88 than in T2288.

Further analysis of soluble sugar components showed that sucrose, glucose, and maltose displayed similar increasing trends with decreasing temperature (Figure 4B,D,E). The absolute levels and relative increases in these sugars were consistently higher in L88 than in T2288. Compared with the control (22 °C), sucrose, fructose, glucose, and maltose contents in L88 increased by 868.28%, 170.26%, 205.55%, and 187.77%, respectively, under −4 °C stress (Figure 4B–E). In T2288, the corresponding increases were 202.14%, 98.62%, 69.83%, and 80.47% (Figure 4B–E). Fructose exhibited distinct cultivar-specific patterns (Figure 4C). In L88, fructose content decreased slightly from 4.66 mg/g at 22 °C to 3.80 mg/g at 4 °C, and then increased continuously as the temperature decreased, rising from 5.64 mg/g at 0 °C to 12.59 mg/g at −4 °C, representing a 123.27% increase. In contrast, fructose content in T2288 peaked at 7.54 mg/g under 4 °C treatment and then declined gradually as the temperature decreased from 0 °C to −4 °C, reaching 5.90 mg/g at −4 °C. At −4 °C, the fructose content in L88 was 6.69 mg/g higher than in T2288.

Overall, from 4 °C to −4 °C, soluble sugar, sucrose, glucose, and maltose contents increased in both cultivars as the stress temperature decreased. However, their contents and increments were greater in L88 than in T2288. Among the soluble sugar components, fructose displayed the most distinct pattern, increasing continuously in L88 but declining in T2288 as the temperature decreased from 4 °C to −4 °C, whereas it gradually declined in T2288.

### 2.5. Transcriptome Profiling of Winter Rapeseed Cultivars with Contrasting Cold Tolerance Under Cold Stress

**Figure 5:**
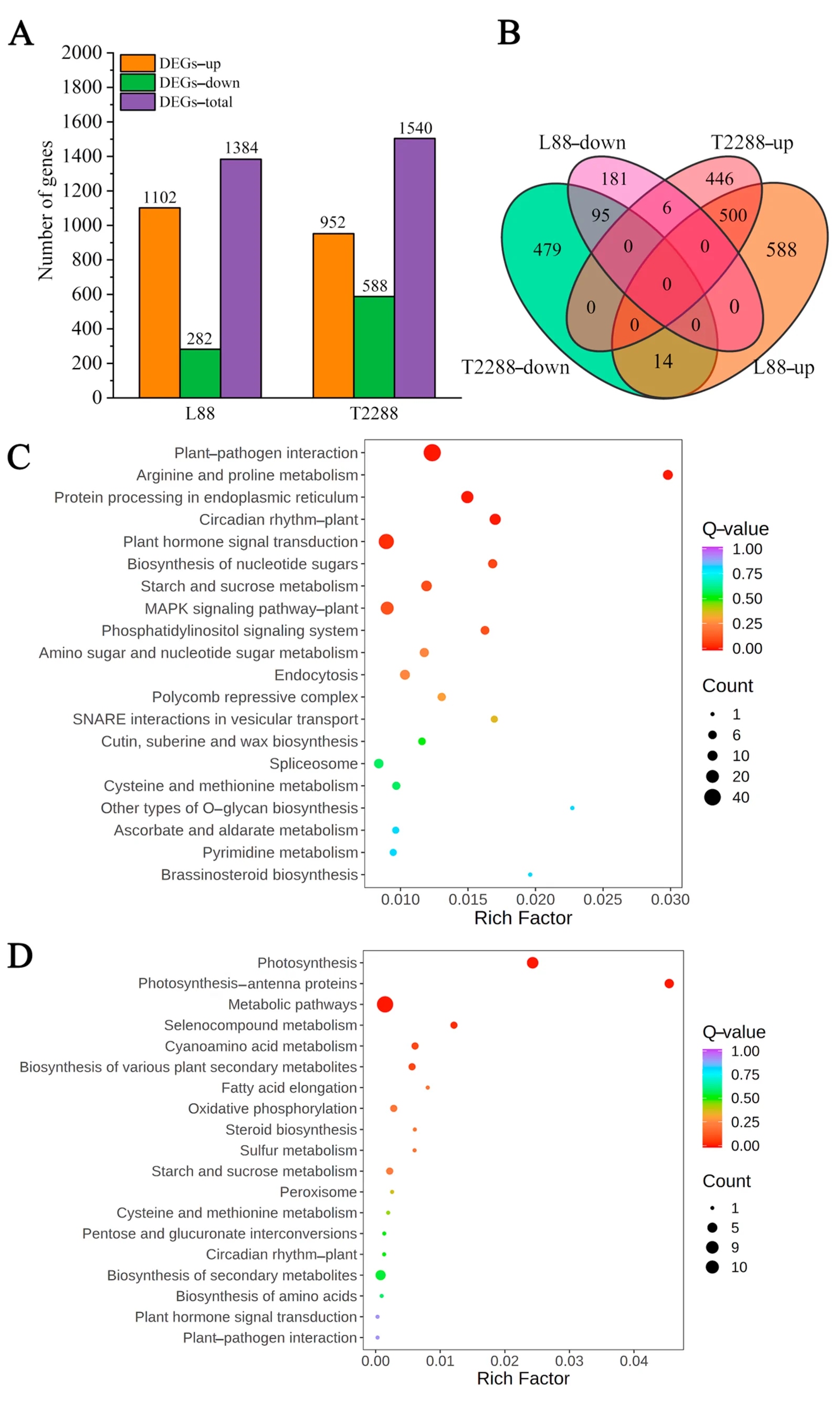
Identification and KEGG enrichment analysis of differentially expressed genes (DEGs) in cold-tolerant winter rapeseed (*B. napus*) L88 and cold-sensitive winter rapeseed (*B. napus*) T2288 under −4 °C cold stress. Leaf samples were collected at 0 h (untreated control) and 24 h after −4 °C treatment for RNA-seq analysis. (**A**) Statistical histogram of upregulated, downregulated, and total DEGs in L88 and T2288; orange bars: upregulated DEGs (DEGs—up), green bars: downregulated DEGs (DEGs—down), purple bars: total DEGs (DEGs—total). (**B**) Venn diagram illustrating overlaps of DEGs with different expression patterns in the two cultivars. L88—up: upregulated DEGs of L88 (orange region); L88—down: downregulated DEGs of L88 (green region); T2288—up: upregulated DEGs of T2288 (pink region); T2288—down: downregulated DEGs of T2288 (purple region). (**C**) KEGG pathways of commonly upregulated DEGs in L88 and T2288; (**D**) KEGG pathways of commonly downregulated DEGs in L88 and T2288.

In this study, we set four temperature gradients (22 °C, 4 °C, 0 °C, and −4 °C) to observe phenotypic, ultrastructural, and physiological changes in the two rapeseed cultivars. The greatest differences in phenotypic starch and soluble sugar contents, as well as chloroplast structural damage, were observed under −4 °C treatment between cold-tolerant L88 and cold-sensitive T2288. Thus, RNA-seq was conducted exclusively on samples collected at 0 h (untreated control) and 24 h after −4 °C cold stress. We analyzed the gene expression patterns of the two cultivars to uncover the molecular basis of winter rapeseed cold tolerance under severe low-temperature stress. Differentially expressed genes (DEGs) were identified using the criteria of |log_2_ fold change| ≥ 1, *p* < 0.05, and fragments per kilobase of transcript per million mapped reads (FPKM) ≥ 10. As shown in Figure 5A, comparison between 24 h cold-stress and 0 h identified 1384 DEGs in L88, including 1102 upregulated and 282 downregulated genes. In T2288, 1504 DEGs were identified under the same comparison, including 952 upregulated and 588 downregulated genes. Comparative analysis further revealed 500 commonly upregulated and 95 commonly downregulated DEGs in both cultivars (Figure 5B).

KEGG pathway enrichment analysis showed that the DEGs commonly upregulated genes were significantly enriched in multiple stress- and metabolism-related pathways, including plant–pathogen interaction, arginine and proline metabolism, protein processing in the endoplasmic reticulum, endocytosis, circadian rhythm–plant, plant hormone signal transduction, biosynthesis of nucleotide sugars, starch and sucrose metabolism, MAPK signaling pathway–plant, phosphatidylinositol signaling system, and amino sugar and nucleotide sugar metabolism (Figure 5C). In contrast, the DEGs commonly downregulated were mainly enriched in photosynthesis, photosynthesis–antenna proteins, selenocompound metabolism, cyanoamino acid metabolism, biosynthesis of various plant secondary metabolites, fatty acid elongation, oxidative phosphorylation, steroid biosynthesis, sulfur metabolism, and starch and sucrose metabolism (Figure 5D).

Among the common DEGs enriched in the starch and sucrose metabolism pathway, 13 genes were identified in both cultivars. Of these, 11 were upregulated, including *BAM1* (*BnaC09g21440D* and *BnaA07g05790D*), *BAM3* (*BnaC07g34180D* and *BnaA03g42940D*), *SCRK1 (BnaCnng30740D)*, *SUS1* (*BnaC09g37040D* and *BnaA10g14710D*), *E1314* (*BnaC03g47100D* and *BnaA03g22360D*), and *TPS9* (*BnaC08g06450D* and *BnaA08g20280D*). The remaining two genes, *BnaA01g27000D* and *BnaC01g34490D*, were downregulated and annotated as *BGL44*.

**Figure 6:**
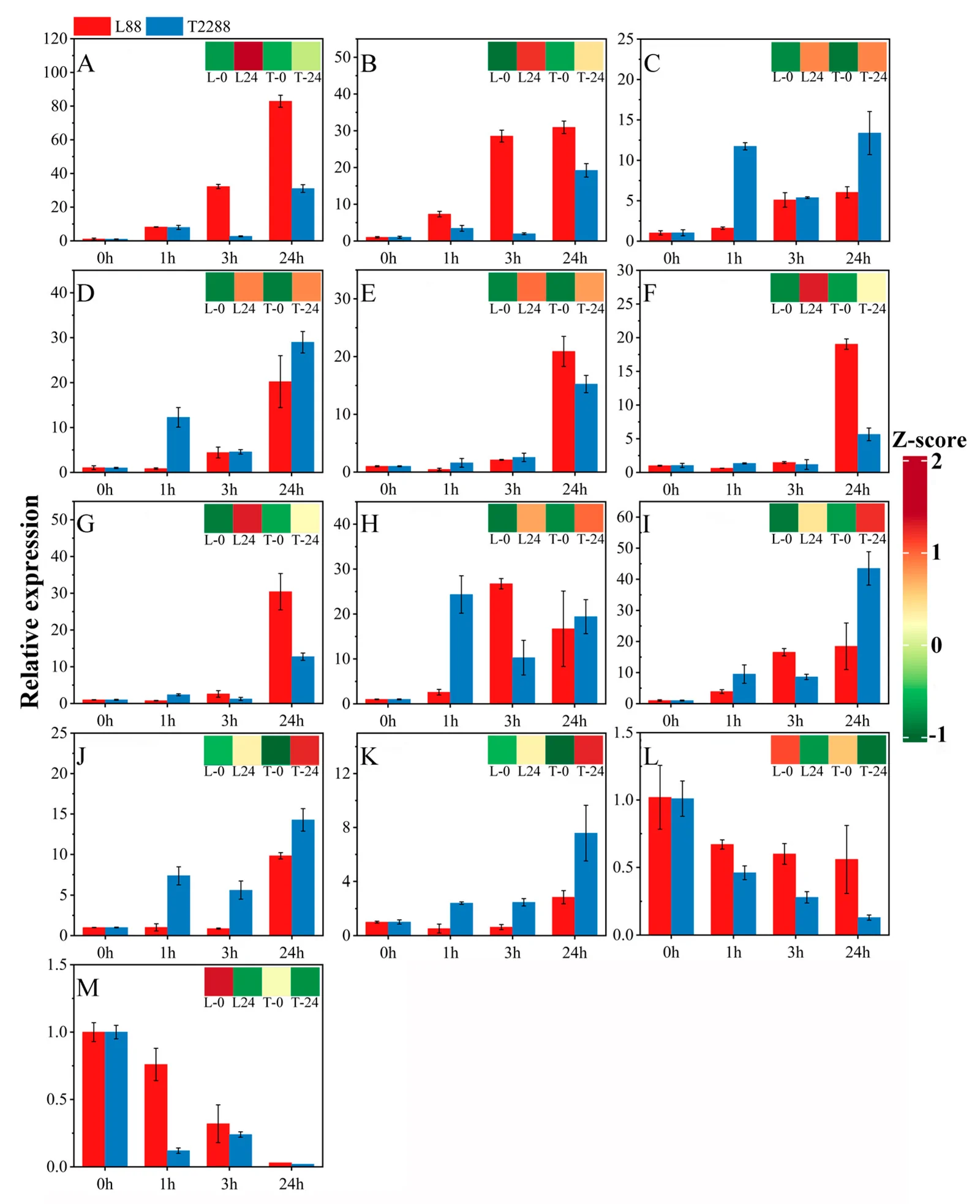
Expression analysis of DEGs involved in the starch and sucrose metabolism pathway based on qRT-PCR and gene clustering heat map. (**A**) *BnaC07g34180D*; (**B**) *BnaA03g42940D*; (**C**) *BnaCnng30740D*; (**D**) *BnaC09g21440D*; (**E**) *BnaA07g05790D*; (**F**) *BnaA10g14710D*; (**G**) *BnaC09g37040D*; (**H**) *BnaC03g47100D*; (**I**) *BnaA03g22360D*; (**J**) *BnaC08g06450D*; (**K**) *BnaA08g20280D*; (**L**) *BnaA01g27000D*; (**M**) *BnaC01g34490D*. Small heat-map insets show transcriptome expression patterns based on Z-score. Abbreviations in heat-maps: L-0, L88 at −4 °C for 0 h; L-24, L88 at −4 °C for 24 h; T-0, T2288 at −4 °C for 0 h; T-24, T2288 at −4 °C for 24 h. The Z-score color scale is presented on the right. Relative expression values are shown as mean ± standard error (SE).

The expression patterns of these 13 genes were further validated by qRT-PCR, and the results were consistent with the gene expression heat map (Figure 6A–M). After 24 h of −4 °C cold stress, *BAM1*, *BAM3*, *SCRK1*, *SUS1*, *E1314*, and *TPS9* were upregulated in both cultivars. Among them, *BAM1*, *BAM3*, and *SUS1* showed strong induction in L88, with approximately 15- to 80-fold increases relative to 0 h; their induction levels ranged from 0.3- to 2.6-fold those in T2288. Conversely, *BGL44* (*BnaA01g27000D* and *BnaC01g34490D*) was downregulated in both cultivars after 24 h cold stress, with transcript levels decreasing by approximately 1.8- to 33.3-fold in L88 and 7.7- to 50-fold in T2288. The extent of BGL44 repression was greater in T2288 than in L88.

## 3. Discussion

### 3.1. Chloroplast Integrity and Starch Degradation Under Cold Stress Are Potential Indicators of Cold Tolerance in Winter Rapeseed

Phenotypic responses to low-temperature stress provide useful visual indicators for evaluating plant cold tolerance. Sun et al. [21] reported that, after exposure to 4 °C and 0 °C for 24 h, the cold-sensitive winter *Brassica rapa* L. (*B. rapa*) cultivar Lenox showed pronounced wilting and leaf drooping, whereas the cold-tolerant cultivar Longyou 7 showed no visible phenotypic changes. Following freezing stress at −4 °C for 24 h, Lenox exhibited more severe freezing injury in leaves than Longyou 7. In the present study, neither of the two winter rapeseed (*B. napus*) cultivars exhibited obvious phenotypic changes after 24 h at 4 °C relative to the control. However, wilting symptoms appeared to varying degrees after 24 h at 0 °C, and water-soaked lesions associated with freezing injury were observed after exposure at −4 °C. These differences may reflect species- and genotype-specific variation in cold tolerance between winter *B. rapa* and winter *B. napus*.

Chloroplasts are among the most cold-sensitive organelles in plant cells, and their ultrastructure is highly susceptible to low-temperature stress. Similar ultrastructural alterations under low temperature have been reported in cotton [22], *Sedum emarginatum* [23], potato [24], and other plant species. In mesophyll cells of *Osmanthus fragrans* ‘Dahua Dangui’, chloroplasts are particularly sensitive to low temperature, and chloroplast stability has been proposed as a key indicator for assessing cold tolerance [25]. In *B. napus*, freezing-sensitive germplasm shows pronounced chloroplast deformation under freezing stress, including disorganization of grana lamellae, disruption of chloroplast membrane structures, and degradation of chloroplast components, whereas cold-tolerant germplasm maintains relatively intact chloroplast structures [26]. Consistent with these findings, our results showed that exposure at 0 °C induced chloroplast swelling in the cold-tolerant cultivar L88, whereas the chloroplast grana lamellae in the cold-sensitive cultivar T2288 became less distinct. Under −4 °C freezing stress, chloroplasts in L88 were swollen and nearly spherical, with a marked reduction in the number of starch granules relative to the control of the same cultivar. In contrast, when compared under this freezing-stress condition, mesophyll cells of T2288 exhibited plasmolysis, chloroplast displacement toward the cell center, and degradation of chloroplast structures. Under cold stress, damage to chloroplast ultrastructure disturbs photosynthetic capacity owing to disordered grana lamellae and impaired thylakoid membranes [8]. Further, the intact subcellular microenvironment required for starch-degrading enzymes is disrupted in injured chloroplasts, which constrains normal starch granule degradation [9]. Consequently, starch cannot be efficiently broken down to supply carbon skeletons and energy substrates for plant physiological processes. In cold-sensitive cultivar T2288, more severe chloroplast structural deterioration further blocks starch turnover, aggravating carbon metabolic disturbance under low-temperature stress.

Together with observations of chloroplast ultrastructure, these findings indicate that the cold-tolerant cultivar L88 maintained relatively intact chloroplast ultrastructure and exhibited more extensive starch degradation under cold stress, whereas the cold-sensitive cultivar T2288 showed severe chloroplast damage and less extensive starch degradation. Therefore, chloroplast integrity and the extent of starch degradation under cold stress may serve as useful indicators for assessing cold tolerance in winter rapeseed.

### 3.2. Association Between Differential Expression of Sugar Metabolism-Related Genes and Cold Tolerance Under Cold Stress

Sugar metabolism is a complex process catalyzed by a series of enzymes. Soluble sugars help maintain cellular membrane stability, and the composition of soluble sugars that accumulate in response to cold stress differ substantially among plant species. In *Arabidopsis thaliana*, sucrose content increases significantly at 4 °C, whereas glucose and fructose contents increase only slightly [27]. In potato, cold acclimation at 4 °C leads to increased levels of total soluble sugars, sucrose, glucose, and fructose in leaves compared with the control [28]. In wheat and rye seedlings, sucrose and fructose contents levels increase rapidly under low-temperature stress [29,30]. In the present study, sucrose and glucose contents increased in both cultivars as treatment temperature decreased. Among the measured soluble sugars, sucrose exhibited the most pronounced increase. Fructose exhibited different accumulation patterns in the two cultivars under low-temperature stress. Fructose continuously increased in cold-tolerant L88, whereas it peaked at 4 °C and subsequently decreased in cold-sensitive T2288. This different pattern is likely associated with cultivar differences in cold tolerance. Under identical temperature-stress conditions, T2288 suffered more severe injury to mesophyll cell and chloroplast structures than L88. Impaired chloroplast function may block timely starch remobilization toward soluble-sugar production, which represents one possible factor contributing to the altered fructose dynamic in T2288.

Transcriptome analysis further revealed that, under cold stress, a greater number of DEGs were upregulated in L88 than in T2288, whereas the number of downregulated DEGs in T2288 was approximately twice that in L88. This indicates that cold stress activated a broader transcriptional response in L88, whereas transcriptional repression was more extensive in T2288. These differences may be attributable to cultivar-specific variation in the magnitude of cold-responsive gene expression, as well as differences in the regulation of sugar metabolism.

*β*-Amylase (BAM) is a key enzyme mediating starch hydrolysis in plant leaves and plays an important role in soluble sugar accumulation under cold stress [31]. Compared with wild-type plants, tomato and *Arabidopsis* plants overexpressing *VaBAM1* exhibit increased amylase activity and soluble sugar content, along with reduced starch content [32]. Overexpression of *PtrBAM1* in tobacco increases BAM activity under low temperature, promotes starch degradation, and enhances the accumulation of maltose and soluble sugars; conversely, silencing *PtrBAM1* in lemon inhibits starch degradation and reduces soluble sugar accumulation [33]. In potato lines with silenced *StBAM1*, BAM activity is reduced and starch content increases [34]. Overexpression of *PbrBAM3* in *Nicotiana benthamiana* and pear promotes BAM activity and starch degradation under low temperature, thereby enhancing plant cold tolerance [35]. In this study, the relative expression levels of *BAM1* (*BnaC09g21440D* and *BnaA07g05790D*) and *BAM3* (*BnaC07g34180D* and *BnaA03g42940D*) increased by more than 15-fold under cold stress, with *BAM3* expression in L88 showing an approximately 80-fold increase. This expression pattern is consistent with the faster starch degradation and greater soluble sugar accumulation observed in L88, suggesting that elevated expression of BAM genes may promote starch degradation and soluble sugar accumulation, thereby contributing to enhanced cold tolerance in winter rapeseed (*B. napus*).

Sucrose synthase (SUS) is a key enzyme in sucrose metabolism. Under low-temperature stress, changes in the expression of sucrose metabolism-related genes modulate sucrose hydrolysis and transport, influence intracellular sugar partitioning and utilization, and thereby improve plant cold tolerance [36]. In the cold-tolerant cabbage cultivar ‘CT-923’, the expression levels of *BoSUS1a* and *BoSUS1b* in leaves increase sharply after 24 h of low-temperature treatment, whereas the magnitude of upregulation in the cold-sensitive cabbage cultivar ‘CS-D9’ is significantly lower than that in ‘CT-923’ [37]. In the present study, *SUS1* (*BnaC09g37040D* and *BnaA10g14710D*) was upregulated in both cultivars under cold stress, with a pronounced increase after 24 h of treatment. However, the relative expression levels of *SUS1* in the cold-tolerant cultivar L88 were much higher than those in the cold-sensitive cultivar T2288, with expression levels approximately 2.0- to 2.6-fold higher in L88 than in T2288. This suggests that *SUS1* is transcriptionally responsive to cold stress and may be involved in SUS-mediated sucrose hydrolysis and sugar metabolism, thereby potentially contributing to the greater cold tolerance of L88 (Figure 6). Collectively, compared with T2288, L88 accumulated more soluble sugars, had a greater number of upregulated DEGs, and showed stronger upregulation of *BAM1*, *BAM3*, and *SUS1*. These factors may partly explain why L88 has superior cold tolerance than T2288.

## 4. Materials and Methods

### 4.1. Plant Materials and Treatments

In this study, Longyou 88 (L88), a cold-tolerant winter rapeseed (*B. napus*) cultivar developed by the Gansu Agricultural University (Lanzhou, Gansu Province, China), and Tianyou 2288 (T2288), a cold-sensitive winter rapeseed (*B. napus*) cultivar developed by the Tianshui Agricultural Science Research Institute (Tianshui, Gansu Province, China), were used as experimental materials.

Healthy seeds were rinsed three times with distilled water and then placed on moist filter paper in Petri dishes for germination under controlled conditions at 25 °C with a 16 h light/8 h dark photoperiod. When the radicles reached approximately 2 cm in length, the seedlings were transplanted into plastic pots containing nutrient-rich substrate, with three seedlings per pot. The plants were grown in a growth chamber under controlled conditions of 22 °C/18 °C (day/night) temperature, a 16 h light/8 h dark photoperiod, and the light intensity of 6000 lx. At the seven-leaf stage, the seedlings were transferred to a low-temperature incubator for cold treatment.

For the cold treatments, seedlings were exposed to 4 °C, 0 °C, and −4 °C for 24 h, with 22 °C used as the control. All low-temperature treatments were maintained under a 16 h light/8 h dark photoperiod with 6000 lx light intensity. The third leaf from the base was harvested, immediately frozen in liquid nitrogen, and stored at −80 °C. All leaf samples of the 0 h control and 24 h cold-stress treatment were collected at 10:00 a.m. uniformly to avoid the influence of sampling time differences on carbohydrate quantification results. Starch, soluble sugar, sucrose, fructose, glucose, and maltose contents were measured using the frozen samples. Fresh leaves were collected for potassium iodide staining and subsequent cell ultrastructural observations. All treatments were performed in triplicate, and representative results are shown.

### 4.2. Physiological Measurement and Cell Ultrastructure Observations

Starch and soluble sugar contents were quantified according to procedures outlined in *Experimental Guide for Plant Physiology* [38]. Sucrose, fructose, and glucose were further measured by corresponding colorimetric protocols from this handbook, while maltose content was determined using a commercial assay kit (Keming Biotechnology, Suzhou, Jiangsu China). Starch accumulation in leaves was visualized by iodine–potassium iodide (*I*_2_–KI) staining as described previously [39]. Ultrathin sections were prepared according to the method of You et al. [40]. Cell ultrastructure observation was performed using a Hitachi HT7800 transmission electron microscope (TEM, Hitachi High-Technologies Corporation, Tokyo, Japan) at an accelerating voltage of 80 kV.

### 4.3. Transcriptome Sequencing and Data Processing

The cold-tolerant cultivar L88 and the cold-sensitive cultivar T2288 were used for transcriptome analysis. Leaf samples were collected at 0 h before treatment and after 24 h of exposure at −4 °C, immediately frozen in liquid nitrogen, and sent to Wuhan Metware Biotechnology Co., Ltd. (Wuhan, China) for RNA sequencing on an Illumina platform.

Total RNA was isolated using TRIzol reagent (Wuhan Metware Biotechnology Co., Ltd., China) and residual genomic DNA was removed with RNase-free DNase (Promega, USA). RNA integrity was evaluated by non-denaturing agarose gel electrophoresis. RNA purity was assessed using a NanoDrop 2000 spectrophotometer (Thermo Fisher Scientific, USA), and RNA concentration and integrity were further determined with an Agilent 2100 Bioanalyzer (Agilent Technologies, USA). Equal amounts of qualified RNA from each sample were used for cDNA library construction with the TruSeq Stranded mRNA LT Sample Prep Kit (Illumina, USA) according to the manufacturer’s protocol. After library quality assessment, the libraries were subjected to paired-end sequencing on the Illumina platform. Raw reads were filtered to remove adapter sequences and low-quality reads containing more than 10% unknown bases. The resulting high-quality clean reads were retained for downstream analyses [41].

### 4.4. qRT-PCR Validation of Differentially Expressed Genes

Thirteen differentially expressed genes screened from the transcriptome data of leaves treated at −4 °C for 24 h (0 h as control) were selected for qRT-PCR validation. The *B. napus* Actin gene was used as the internal reference gene. Specific primers were synthesized by Sangon Biotech Co., Ltd. (Shanghai, China), and their sequences are listed in Table 1. Total RNA from the two cultivars were extracted using a total RNA extraction kit (DP452, Tiangen Biotech, Beijing, China). First-strand cDNA was synthesized using a PrimeScript RT reagent Kit (RR036A, TaKaRa, Beijing, China) following the manufacturer’s instructions. qRT-PCR was performed with FastReal qPCR PreMix (FP217, Tiangen Biotech, Beijing, China) according to the manufacturer’s protocol. Each sample was analyzed in three biological replicates, and relative gene expression levels were calculated using the 2^−ΔΔCt^ method [42].

### 4.5. Statistical Analysis

Data were organized using Microsoft Excel (version 24.0; Microsoft Corporation, Redmond, WA, USA) and statistically analyzed using SPSS Statistics (version 24.0; IBM Corp., Armonk, NY, USA). Independent sample *t*-tests were performed, and asterisks indicate significant differences: ns represents not significant (*p* ≥ 0.05), * represents *p* ≤ 0.05, ** represents *p* ≤ 0.01, and *** represents *p* ≤ 0.001. Figures were prepared using Origin (version 2024.0; Origin Lab Corp., Northampton, MA, USA) and Adobe Photoshop (version 24.0; Adobe Inc., San Jose, CA, USA).

## 5. Conclusions

Under cold stress, the cold-tolerant Longyou 88 maintained better chloroplast integrity, exhibited more extensive starch degradation and greater soluble sugar accumulation, and showed stronger transcriptional activation of starch- and sucrose-metabolism-related genes than the cold-sensitive Tianyou 2288. In contrast, Tianyou 2288 exhibited more severe chloroplast damage, retained more undegraded starch granules, and had approximately twice as many downregulated DEGs as Longyou 88. Among the DEGs enriched in the starch and sucrose metabolism pathway, *BAM1*, *BAM3*, and *SUS1* showed greater upregulation in Longyou 88. These results suggest that chloroplast integrity, starch degradation, soluble sugar accumulation, and the cold-induced expression patterns of *BAM1*, *BAM3*, and *SUS1* may be associated with cold tolerance and could serve as potential indicators for evaluating cold tolerance in strong-winter-type winter *B. napus* in northern China. These observations advance our understanding of carbohydrate-mediated cold-acclimation responses and offer useful references for local rapeseed breeding programs. Nevertheless, several limitations should be noted. Only two cultivars were examined in this study, transcriptome analysis was restricted to −4 °C treatment, and the biological functions of *BAM1*, *BAM3*, and *SUS1* remain unvalidated experimentally. Future work will focus on functional verification of these candidate genes to establish reliable selection criteria for cold-tolerant winter rapeseed.

## Figures and Tables

**Table 1:** The primer sequence for qRT-PCR.

Gene Name	Forwards Sequence (5′-3′)	Reverse Sequence (5′-3′)
*BnaC07g34180D*	GGGAATGGGACCTTGTGGAG	GAGGTCCACTTGTTCCCCAG
*BnaA03g42940D*	CTCATGACGCCGGAGAGTAC	TTGGCTGAAGACAGGAGCTG
*BnaCnng30740D*	TCCGTTGACCCTTTCCATGG	CGAGGATGGAGTGGTCATCG
*BnaA10g14710D*	TCACCTCTCGGCTAAGCTCT	TGCCAGATACTCCTCTGCCT
*BnaC09g21440D*	TTAGCCAGACACAACGCCAT	GCCACCTGGTTCACTAGCTT
*BnaC09g37040D*	TCCGTTGACCCTTTCCATGG	CGAGGATGGAGTGGTCATCG
*BnaA07g05790D*	GAACAGAAACGTATCGCCGC	CGAACACAGGAACTCCTCCC
*BnaC03g47100D*	CTTGTTGCGGTTGGTTCAGG	CTCTCAGAAATCGGACCCGG
*BnaA03g22360D*	GGATCCAGCAACGACTCCAA	ACCGAGACTGACCAAAGCAG
*BnaC08g06450D*	AGAGATATTCGCCTGCACGG	AAACGCCACATGTGTTCGTG
*BnaA08g20280D*	TACGGCTCCCCTGGTTATGA	GTCTCTTACCGCGTTCACCA
*BnaA01g27000D*	TATTCGCACCGGGTAGATGC	TGCTGCATGGGCCAAGATAA
*BnaC01g34490D*	TATTCGCACCGGGTAGATGC	TGCTGCATGGGCCAAGATAA
*ACTIN*	TGTGCCAATCTACGAGGGTTT	TTTCCCGCTCGGCTGTTGT

## Data Availability

The datasets used and/or analyzed during the current study are available from the corresponding author on reasonable request. The transcriptomic sequencing data can be consulted via the reviewer-only link: https://dataview.ncbi.nlm.nih.gov/object/PRJNA1478463?reviewer=dkb90cu32bigssct2fsdcf0265. The raw sequence data will be publicly released on 1 August 2027.
